# Healing grief: Insights from relatives of cancer patients with prolonged grief. The FamiLife multicenter qualitative study

**DOI:** 10.1017/S1478951524001068

**Published:** 2024-10-31

**Authors:** Cécile Flahault, Léonor Fasse, Laetitia Veber, Marie Sonrier, Marie Annick Leborgne, Dominique Michel, Véronique Marché, Anne Vanbésien, Adrien Evin, Nicolas Pujol, Laure Copel, Willeme Kaczmarek, Sylvie Kirsch, Catherine Verlaine, Virginie Verliac, Emmanuel Delarivière, Virginie Fosset-Diaz, Virginie Guastellas, Véronique Michonneau-Gandon, Ségolène Perruchio, Gaelle Ranchou, Laurence Birkui de Francqueville, Cécile Poupardin, Licia Touzet, Carmen Mathias, Alaa Mhalla, Guillaume Bouquet, Bruno Richard, Dominique Gracia, Florent Bienfait, Stéphane Ruckly, Jean François Timsit, Maité Garrouste-Orgeas

**Affiliations:** 1 Paris-Cité University Paris, Laboratory of Psychopathology and Health Process UR4057, Boulogne-Billancourt, France; 2 Palliative Care Unit, Reuilly Diaconesses Fondation, Rueil Malmaison, France; 3 Palliative Care Unit, Cognacq-Jay Hospital, Paris, France; 4 Palliative Care Unit, General Hospital, Douai, France; 5 Palliative Care Unit, University Teaching Hospital, Nantes, France; 6 Research Department Palliative Care Unit, Jeanne Garnier Institution, Paris, France; 7 Palliative Care Unit, Diaconesses Croix Saint Simon Hospital, Paris, France; 8 Palliative Care Unit, La Dracénie Hospital, Draguignan, France; 9 Palliative Care Unit, Bligny Hospital, Briis-Sous-Forges, France; 10 Palliative Care Unit, General Hospital, Troyes, France; 11 Palliative Care Unit, Saintonge General Hospital, Saintes, France; 12 Palliative Care Unit, Marie Galène Institution, Bordeaux Caudéran, France; 13 Palliative Care Unit, Bretonneau Hospital, Paris, France; 14 Palliative Care Unit, University Teaching Hospital, Clermont Ferrand, France; 15 Palliative Care Unit, Castres-Mazamet General Hospital, Castres, France; 16 Palliative Care Unit, Rives de Seine Hospital, Puteaux, France; 17 Palliative Care Unit, General Hospital, Périgueux, France; 18 Palliative Care Unit, Compiègne Noyon Hospital, Compiègne, France; 19 Palliative Care Unit, General Hospital, Montfermeil, France; 20 Palliative Care Unit, University Teaching Hospital, Lille, France; 21 Palliative Care Unit, Mulhouse Sud Alsace Hospital Network, Mulhouse, France; 22 Palliative Care Unit, Albert Chenevier Hospital, Créteil, France; 23 Palliative Care Unit, Tourcoing General Hospital, Tourcoing, France; 24 Palliative Care Unit, University Teaching Hospital, Montpellier, France; 25 Palliative Care Unit, General Hospital, Salon-de-Provence, France; 26 Palliative Care Unit, University Teaching Hospital, Angers, France; 27 Biostatistical department, ICURESEARCH SAS, Paris, France; 28 AP-HP, Bichat Hospital, Medical and infectious diseases ICU (MI2), Paris, France; 29 Université de Paris, IAME, INSERM, Paris, France; 30 Medical Unit, French British Hospital, Levallois-Perret, France

**Keywords:** Grief, qualitative research, palliative medicine, family, interview

## Abstract

**Background:**

Prolonged grief is a chronic and debilitating condition that affects millions of persons worldwide. The aim of this study was to use a qualitative approach to better understand how relatives with prolonged grief disorder perceive what does or not help them and whether they were able to make recommendations.

**Methods:**

Participants were all relatives of deceased patients admitted to 26 palliative care units involved in the FamiLife study; relatives were included if diagnosed with prolonged grief symptoms (i.e., Inventory Complicated Grief (ICG) questionnaire with a cut-off >25), and volunteered to participate. Semi-directed telephone interviews were conducted by psychologists between 6 and 12 months after the patient’s death. The interviews were open-ended, without a pre-established grid, then transcribed and analyzed using a thematic approach.

**Results:**

Overall, 199/608 (32.7%) relatives were diagnosed with prolonged grief symptoms, i.e., with an ICG score >25, and 39/199 (20%) agreed to be interviewed. The analysis yielded 4 themes: (1) the experience of mourning: intense sadness and guilt (reported by 35/39 participants, 90%); (2) aggravating factors (38/39, 97%): feeling unprepared for death and loneliness, presence of interpersonal barriers to adjustment, external elements hindering the mourning progress; (3) facilitating factors (39/39, 100%): having inner strength or forcing oneself to get better, availability of social and emotional support; and (4) the suggestions grieving relatives had to alleviate the grief burden (36/39, 92%). The analysis enabled to identify 5 suggestions for relieving the grief burden: improving communication, developing education about death and grief, maintaining contact, offering psychological support, and choosing the right time for the palliative care team to contact the relatives.

**Conclusions:**

This study revealed how bereaved relatives experienced the help provided by the healthcare teams, their representations, and what could be improved. These findings could be used to design intervention studies.

## Introduction

Prolonged grief is a defined chronic and debilitating condition that affects millions of people worldwide. The loss of a close relative is one of the most stressful situations a person can face in life. However, after an acute loss, the majority of persons have normal reactions and are able to return to their normal lives within a few months (Ringdal et al. 2001). Normal grief reactions include a period of sadness, numbness, and even guilt and anger. Gradually these feelings subside, and it becomes possible to accept the loss and to live life and function with potentially having surges of grief in special events. Many symptoms of normal grief and prolonged grief are similar; but, while normal grief symptoms gradually start to fade over time (months, or even years), symptoms of prolonged grief persist or get worse. Prolonged grief is like being in a constant, heightened state of mourning that prevents you from healing and impairs your ability to function in daily life. About 10% people experience prolonged grief symptoms (Szuhany et al. 2021) and do not adjust to the loss of their loved one, develop disbelief or inability to accept the loss and struggle to imagine how they are going to live without the deceased persons (Prigerson et al. 2009; Shear et al. 2013). At least 3 of the following symptoms must be present to a clinically significant level on most days: disruption of identity, marked sense of disbelief about the death, avoidance of reminders that the person is dead, intense emotional pain (such as anger, bitterness, sorrow) related to the death, difficulty reintegrating into their own life, emotional numbness, feeling that life is meaningless, intense loneliness, and characterized by its intensity and duration: 1 year, or 6 months (Szuhany et al. 2021). Prolonged grief reactions at 8 months were reported in 24.4% (Chiu et al. 2010), and at 6 months in 25.4% (Newson et al. 2011), 28.6% (Coelho et al. 2015), 30% (Wiese et al. 2010), and 40% (Guldin et al. 2012) among relatives of a heterogeneous population of cancer patients.

Risk factors associated with the development of prolonged grief reactions include pre-loss risk factors (female gender, pre-existing trauma such as childhood trauma, previous loss, insecure attachment, pre-existing mood and anxiety disorders, nature of the relationships) (Fujisawa et al. 2010; Kersting et al. 2011; Newson et al. 2011) loss-related factors (relationships of caregivers assuming roles, nature of the death itself) (Fujisawa et al. 2010; Kersting et al. 2011; Neria et al. 2007; Newson et al. 2011), and peri-loss factors (social circumstances, resources available after death, poor understanding of the circumstances of death, interference between natural healing process and cultural practices of death) (Bui et al. 2013; Hargrave et al. 2012; Mutabaruka et al. 2012). Although grief severity symptoms decreases over time without specific intervention (Ringdal et al. 2001), its expression varies widely among individuals, circumstances of death and cultures (Silverman et al. 2021).

Prolonged grief reactions must be distinguished from depressive disorders or post-traumatic stress disorder, although there are overlapping features. Prolonged grief disorder is a distinct diagnosis because the criterion is a loss. Anxiety, intrusive memories, hyperarousal are related to the post-traumatic stress disorder induced by the traumatic event (Szuhany et al. 2021). Symptoms of depressive disorders include in part reduced interest in or enjoyment of everyday activities, not automatically focused on the deceased (Kristensen et al. 2017).

In France, bereavement support for relatives is left to the discretion of the palliative care units in the absence of specific recommendations. Only the practices specific to each service are documented, without necessarily knowing how the bereaved themselves might appreciate them. The most common approaches used are either psychological follow-up contacts or sending a letter of condolence, as in other countries (Hayward et al. 2016; Morris et al., 2015).

The aim of this study was to conduct qualitative research to better understand how relatives with prolonged grief symptoms perceive which practices are helpful or not in alleviating grief, and whether they are able to provide recommendations for pre- and post-loss management.

## Methods

### Participants

This study was part of the prospective observational multicenter mixed study (FamiLife) conducted in 26 palliative care units in France (recruitment period from January 2019 to February 2020). Participants were relatives of deceased patients diagnosed with a neoplasia and expected to be hospitalized for more than 72 hours for end-of-life issues. Participants were recruited after their relative died. Follow-up was completed in August 2020.

The primary outcome was the incidence of prolonged grief symptoms, defined by an Inventory Complicated Grief (ICG) score >25 (0 best–76 worst) assessed 6 months after the patient’s death (Maciejewski et al. 2016; Zisook et al. 2010). Prespecified secondary outcomes were the risk factors for prolonged grief symptoms, anxiety and depression symptoms between day 3 and day 5 and 6 months after the patient’s death, based on the Hospital Anxiety and Depression Score (Zigmond and Snaith 1983) (range 0–42) >8 for each subscale, post-traumatic stress disorder symptoms 6 months after the patient’s death based on the Impact of Events Scale questionnaire (Rash et al. 2008) (0 best–88 worst) score >22.

One of the secondary objectives was to understand the complex system of grief. Between 6 and 12 months after the patient’s death, a phone interview with relatives with prolonged grief symptoms (IGC > 25) was planned. The 6-months period was chosen because grief reactions decrease as a function of time after death, with the steepest decrease from the first month to the sixth month after death, while the decrease is no longer significant from 6 months to 13 months (Guldin et al. 2012; Ringdal et al. 2001). The protocol and the quantitative part have been published elsewhere (Garrouste-Orgeas et al. 2019; Garrouste-Orgeas et al. 2023). The quantitative results of FamiLife study showed that 6 months after the death of the cancer patients, 611surveyed relatives reported: prolonged grief symptoms (32.7%), post-traumatic stress disorder symptoms (53.2%), anxiety (37.7%), and depression (18.1%).

### Data collection and analyses procedures

Relatives involved in the study came from all centers (Table 1). There was no relationship between participants and researchers prior to the first telephone call. Semi-structured interviews were conducted by telephone by 3 female psychologists (CF, MS, MAL) specifically hired for the study. They were all familiar with the care of patients with severe physical conditions, and with qualitative bereavement research. Each participant completed only 1 interview. The participants knew the role, training and commitment of the researchers on the topic of the mourning, which was their main research topic. The interview began with the instruction: “In the previous phase of the study, you described difficulties following the death of your loved one. Could you tell us more about these and what do you think helped you or is helping you, or, on the contrary, what was a major difficulty.” No pre-established grid was used, but rephrasing was used to encourage relatives to go deeper into their experience of grief. The purpose of the interviews was to collect the widest variety of facilitators and barriers to grief healing. Data saturation was reached for the major themes reported.

**Table 1.** Characteristics of eligible and interviewed relatives

Variables	Relatives eligible ICG > 25 (*n* = 199)	Relatives not interviewed (*n* = 160)	Relatives interviewed ICG > 25 (*n* = 39)	Missing data	*p*-value
Demographics					
Age, median (IQR), y	57 [42; 67]	57 [41; 67]	57 [46; 68]	10	0.83
Women gender, %	155 (77.9)	127 (79.4)	28 (71.8)	0	0.39
Relationship with the patient, %				13	0.96
Spouse/partner	89 (47.8)	70 (47.3)	19 (50)		
Children	75 (40.3)	59 (39.8)	16 (42.1)		
Parents	9 (4.8)	8 (5.4)	1 (2.6)		
Other	13 (7)	11 (7.4)	2 (5.3)		
Occupation^a^, %				1	0.55
Primary	1 (1)	1 (1.3)	0		
Secondary	6 (6.1)	5 (6.4)	1 (4.8)		
Tertiary	84 (84.8)	67 (85.9)	17 (81)		
None	8 (8.1)	5 (6.4)	3 (14.3)		
Retired, %	79 (79.8)	63 (77.8)	16 (88.9)	0	0.52
Educational level, %				7	0.09
Upper secondary education	21 (10.9)	19 (12.3)	2 (5.4)		
High school	31 (16.1)	23 (14.8)	8 (21.6)		
Technical college	18 (9.4)	18 (11.6)	0		
Associate degree	42 (21.9)	36 (23.2)	6 (16.2)		
Bachelor degree	36 (16.1)	27 (17.4)	9 (24.3)		
Master degree	44 (22.9)	32 (20.6)	12 (32.4)		
Previous palliative care hospitalization for a closer relative, %	41 (20.6)	39 (24.4)	2 (5.1)	0	0.01
Patient’s surrogate, %	127 (63.8)	100 (62.5)	27 (69.2)	0	0.46
Primary caregiver, %	135 (67.8)	109 (68.1)	26 (66.7)	0	0.85
Presence at home, %					
Health-care worker	98 (49.2)	78 (48.8)	20 (51.3)	0	0.86
Life support person	17 (8.5)	13 (8.1)	4 (10.3)	0	0.75

ICG = Inventory Complicated Grief; IQR = Inter Quartile Range.

a Occupations were grouped onto agricultural (primary), industrial (secondary) or commercial or related to administration or human health or public action (tertiary).

In our study, transcription of the interviews was performed by the researchers who interviewed the participant. Once transcribed, the verbatim content was subjected to thematic analysis using the CAQDAS NVivo 10 package (QSR international, Victoria, Australia). Coding followed Braun and Clarke’s recommendations for thematic analysis (Braun 2006), as described in a 6-step guide: (1) becoming familiar with the data, (2) generating initial codes, (3) searching for themes, (4) reviewing themes, (5) defining and naming themes (using the coded data and against the entire data set), and (6) writing a detailed description of the results. Coding, manual identification of themes and sub-themes, and interpretation of results were conducted by a research psychologist trained in qualitative analysis (LV) supervised by 2 senior researchers in psychology trained in qualitative methods (CF, LF), and then discussed within the research team, in order to minimize potential interpretative bias and stay close to the data. The original verbatims were translated into English; names were not included for confidentiality reasons. Transcripts were not returned to participants for comments or corrections. Participants did not provide feedback on the findings.

We complied with the Consolidated Criteria for Reporting Qualitative Research and the COREQ checklist (Tong et al. 2007).

## Results

### Participants

Prolonged grief symptoms, identified by a score >25 of the ICG questionnaires, was reported in 199/608 (32.7%) relatives (e-Table 1). Of the 199 relatives with an ICG score >25 at 6 months after the patient’s death, 39 (20%) gave consent to be interviewed 6 months to 1 year after the patient’s death (e-Table 1). The mean duration of the interviews was: 27.4 ± 10.5 minutes. Characteristics of eligible and included subjects in the qualitative study are shown in Table 1. Participants and non-participants did not differ except for their previous experience of having a close relative hospitalized in a palliative care unit, which was higher among non-participants. Complicated grief symptoms co-occurred with post-traumatic stress disorder in 38/39 (97.4%), anxiety symptoms in 21/39 (53.8%) and depression symptoms in 16/39 (41%).

### Main findings

#### Themes

Four themes emerged from the analysis: (1) the experience of mourning (reported by 35/39 subjects, 90%), (2) the factors that aggravated the experience (38/39, 97%), (3) the factors that facilitated the experience of grief (39/39, 100%), and (4) the suggestions that bereaved relatives may have for good practices to alleviate the grief burden (36/39, 92%) (Table 2).

**Table 2.** Themes and sub-themes

Themes	Sub-themes
**Theme 1**: The experience of mourning: intense sadness and guilt	Intense Sadness that cannot be communicated Guilt and regrets Grief haunted by the burden of the illness experience
**Theme 2**: The worsening factors: what makes grief even more difficult to deal with?	Grief worsened by the feeling of being unprepared for death and loneliness Grief hindered by interpersonal barriers to adjustment External elements hindering the mourning progress: calendar and pandemic
**Theme 3**: The facilitating factors: some elements contribute to alleviating grief	Inner strength or forcing oneself to recover Social and emotional support
**Theme 4**: Suggestion made by the bereaved for better coping with their grief	Improving communication with the medical team Developing education on death and grieving Maintaining bounds Provision of psychological support

##### Theme 1: The experience of mourning: intense sadness and guilt

“The experience of mourning” includes psychological and somatic symptoms that the bereaved may experience but do not always communicate about. These experiences were not reported by all the subjects, who probably focused more on what helped or hindered their grief process.

1. *Intense Sadness that cannot be communicated*

Symptoms of negative mood such as anhedonia were identified. Subjects expressed their lack of energy and motivation and their feelings of emptiness. Verbalization of the loss and social communication were difficult.

Many participants (35/39, 90%), such as (female gender, age 49, daughter) described their inability to communicate:

*And I wasn’t even able to … to … just give news to my friends… uh … or to my family… it required … yes it was uh … it was actually beyond my strength.*

They reported constant and extreme sadness, excessive crying and social isolation. This constant negative mood can lead to dark thoughts and suicidal thoughts (4/39, 10%). For example, (female gender, age 59, daughter) and (female gender, age 59, spouse) said:

*I’d like to stop everything. Nothing would happen anymore and … well, I had a good life, so if it stops today, it’s not a big deal. It doesn’t matter to me (she insisted). It doesn’t matter to me!*

*If I had been alone, without children and none around, I am not sure I would not have tried to end my life. If I had no one, I would have told myself: what’s the point of carrying on living. I think … if I would have had the courage afterwards …, but the idea was truly there.*

Feelings of helplessness/hopelessness permeated the words of (daughter/ female gender, age 59, daughter) and (female gender, age 59, spouse) and lead to a loss of meaning in life after the death of their loved ones.

1. *Guilt and regrets*

Most participants (33/39, 85%) wee haunted by the idea that they did not do everything they could for the patient; whether it was not being present enough at the hospital, not doing enough to ease the pain during the patient’s last days, or not telling their loved ones everything they would have liked to:

*(Male gender, age 75, partner) It’s a feeling I have frequently. I feel like I didn’t do everything I could to make her smile a little bit more …*

*(Female gender, age 53, daughter) so we regret that we got upset like that, maybe I wasn’t the best person to calm her before she left.*

Guilty thoughts are invading their daily life:

*(Female gender, age missing, daughter) I had these images of me dragging my father like an old horse to the slaughterhouse …*

*(Female gender, age 53, daughter) I went through a phase where I really felt guilty, and I cried a lot in the evening, and I told her that she had to forgive me… because I was the one who had poisoned her with all of these drugs while I thought I was curing her …*

These words illustrate the feeling of intense responsibility. Some participants sometimes engage in an imaginary dialog with the deceased, a dialog in which the deceased never responds, and therefore the guilt remains.

1. *Grief haunted by the burden of the illness experience*

The patient’s pain was described as psychologically unbearable for relatives. It causes significant distress, sadness and obsessive thoughts that persist after the loss (35/39, 90%).

*(Male gender, age 53, son) well, uh … well, I have images of my mother in pain that keep coming back to me. […] this episode, when it comes back to me at night before I fall asleep, uh … it can last … ten minutes, a quarter of an hour, and I see the whole “film” all over again*

*(Female gender, age 38, daughter) I have an image that haunts me (insists on the word) […] We found her on the armchair in her room, and that’s it! she wasn’t talking anymore … she was shaking … and that made me … (silence) … […] It’s been, it’s been hard, yeah!*

These words evoke the traumatic nature of some memories. These 2 participants may re-experience traumatic memories of the end of their loved one’s life (shocking symptoms, especially physical pain).

Some participants (20/39, 51%) had to assume medical and emotional responsibilities that further developed their anxiety and obsessive thoughts. These experiences were identified as traumatic and stressful decisions or behaviors they had to perform (administering high doses of morphine, contributing to euthanasia decision, supporting the patient during the dying process …).

*(Male gender, age 71, partner) A very painful thing for me … it was … I had to decide to proceed the medically assisted euthanasia … And … then, I fell apart.*

It must be noted that the healthcare providers did not ask this man to provide euthanasia for his/her relative, but it was his/her reconstruction of that experience.

*(Female gender, age 52, spouse) they forced me to take decisions that I was unable to take … It was Sunday, they had this question about the mortuary chamber, … you see … nobody was there, we couldn’t take the body out, so they asked me if I wanted the body to stay in the room or not … technical questions … it was beyond what I could answer …*

##### Theme 2: Aggravating factors: what makes grief more difficult to deal with?

“Aggravating factors” include thoughts, emotions, and behaviors that negatively influence the grieving process. They are induced by the bereaved him/herself or imposed by social relationships such as family, friends, caring team, and administration.

1. *Grief worsened by feeling unprepared for death, and loneliness*

According to the participants (38/39, 97%), the feeling of loneliness and emptiness is triggered by the violence of the decline and the feeling that life is collapsing. The intensity of these symptoms depends on the closeness of the relatives to the deceased. The abruptness with which the patient’s decline and death occurred contributed to making the last days a traumatic experience. This helpless distress has also been exacerbated by the fact that relatives have felt unprepared for these events.

*(Female gender, age 49, daughter) That’s the thing! It was quite traumatic, I mean, he left very, very quickly …*

*(Female gender, age 71, spouse) I didn’t know what it was … he started to shake, to … I didn’t know what to do … (voice shaking) we’re not uh … I wasn’t prepared to see him like this, at all. (crying)*

Relatives explained how stressful it is to see one’s entire life collapse in a very short period of time. In addition, some participants (20/39, 51%), such as (female gender, age 71, spouse), reported how distressed and shocked they were to witness the physical deterioration of their loved one.

Moreover, Participants (14/39, 36%) expressed that they were suddenly torn away from their daily routine organized around the patient, the treatments and the hospital. This contributed to the development of feelings of loneliness:

*(Female gender, age 52, spouse) because it really looks like a break-up afterwards, I mean, at the hospital the doctor, everyone, is trying to help you … […] And after, well there is nothing, nothing more … there are just you and your distress.*

And as they give up their caregiving role, they also lose their purpose in life; especially in the case of chronic illness:

*(Male gender, age 81, partner) I didn’t have to go to hospital to see my wife anymore, it wasn’t … it wasn’t positive at all … From that moment on, I lost my entire life purpose …*

*(Female gender, age 50, daughter) When the stay in the hospital has been long, you actually realize that when there was someone to look after, there were people around. But afterwards, there is nothing left.*

*(Female gender, age missing, daughter) Because I was looking after him all the time, because we were so close, I might have experienced a kind of emptiness in my life …*

Relatives suffer greatly from the absence of the deceased (30/39, 77%). The closer they were to the patient, the more acute the absence. Even the support of friends and family does not compensate for this feeling of loneliness:

*(Female gender, age 69, spouse) Even though people are around, you feel lonely. That’s another difficult thing of this grieving.*

They also expressed feeling abandoned by the medical team (29/39, 74%). Several participants mentioned their bitterness at what they perceived as a lack of medical support and poor communication from the medical team.

Explanations of the patient’s medical condition are reported to be sporadic, partial, unclear or are simply absent:

*(Male gender, age 53, son) people give you explanations but without really explaining anything, you see? You are meeting with the doctor, but the doctor, with his doctor’s pledge, gives you … sometimes few details, sometimes … nothing … on purpose? or not! I don’t know*

They feel disregarded:

*(Female gender, age 53, daughter) they ask us to leave the bedroom! the doctors! But we are here, and this is our parent or our child: we have the right to hear what the doctors say … there shouldn’t be medical secrecy at this point.*

*(Female gender, age 53, daughter) … well … when I was talking to the doctor, I had the feeling … well … that she was always asking me to shut up, basically. She wanted to talk to my mother, not me.*

They lacked a personalized and empathetic approach; they denounced a lack of communication, especially during the transition from one unit to another:

*(Female gender, age 35, daughter) there was the transition from the hospital day unit to … to the palliative unit[…] but the nurse who picked him up, if I may say, she didn’t really know what was wrong with him […] his medical dossier etc …*

*(Male gender, age 53, son) we always had excellent relationship with her doctor who took care of her for years, but on D-day, typically, was he afraid or not? I don’t know, but it’s another doctor who took me aside and explained to me that, … That’s it! Then you just have your eyes to cry …*

1. *Grief hindered by interpersonal barriers to adjustment*

Many participants (35/39, 90%) explained that they were unable to continue seeing a therapist because:

- They tried to make contact, but no one was reachable:

*(Female gender, age 53, daughter) I tried to be in contact with this psychologist and she was unreachable twice, so I stopped trying …*

- They could not to come back to the hospital to meet with the psychologist:

*(Female gender, age 52, spouse) And well, I think it’s not possible to see this psychologist, I mean, the psychologist who I met during that time … (Silence) in fact, for me, it was not possible, because I couldn’t, I couldn’t … come back …*

*Nothing else was suggested …, except to come back to that place …*

- Or they did not dare ask for help:

*(Female gender, age 35, daughter) I thought about seeing someone to talk about it, but I don’t like to cry in front of others. I was almost ashamed to say to myself: you talk about your life, you cry!*

Some relatives expressed a distress associated with feeling better and mentioned a feeling of “loyalty” to the deceased:

*(Male gender, age 71, partner) I feel guilty to get better and satisfied about getting better! […] That’s it. And when I’m happy, I feel guilty for being happy, because it’s not normal. […] I find it unbearable to go on living while she is gone.*

1. *External elements hindering the mourning progress: the calendar and the pandemic*

Some factors seem uncontrollable even though they aggravate the mourning process (15/39, 38%).

Anniversaries are one of them. As explained by (female gender, age 50, daughter): “*it triggers emotion, feelings of … my life before the death …*”

COVID-19, which appeared at the very end of the recruitment phase, has also increased social isolation and hindered the adjustment to the loss.

*(Female gender, age missing, spouse) The crisis obviously did not help. I was just starting to see my close friends again and the lockdown happened. We were isolated. Obviously like everyone else, but me probably more than others.*

As mentioned by *(female gender, age missing, spouse)*, the lockdown prevented relatives from relying on their friends during this period, marked by a double loneliness: that of the grief and that of the epidemic.

##### Theme 3: The facilitating factors: some elements contribute to alleviating grief

The following theme refers to all the supportive elements, either initiated by the bereaved themselves or by the people around them, including the medical team, mental health professionals and friends, family and close relationships.

1. *Inner strength or forcing oneself to recover*

The first subtheme covers the behavioral and cognitive inner strength that bereaved people used to reduce the pain of loss and to force themselves to recover.

In most cases (36/39, 92%), the bereaved described the beneficial outcome of keeping themselves busy:

*(Female gender, age 69, spouse)*

Keeping oneself busy, proactively trying to recover, often seems to be the only alternative to suicide:

*(Male gender, age 60, partner) And I said to myself from the beginning […] either you shake it off or – crudely – you just have to shut yourself down.*

Participating in this research study was also mentioned as one of the facilitating factors for mourning (30/39, 77%). It helped them to:

- Verbalize: *(Male gender, age 81, partner) You helped me to put into words …*
- Reflect: *(Female gender, age 50, daughter) it’s been a while since I haven’t asked myself questions … haven’t clarified to myself … so, honestly, it’s good to do it.*
- And expressing their feelings: *(Female gender, age 49, daughter) Well it’s always painful to talk about this, but I think it’s necessary … so I’m glad I did it!*

For some of the relatives (16/39, 41%), it was the first time they had talked so deeply about their grief: *(Female gender, age 49, daughter) When I was talking to your colleague for example, well […] I think she was the first person I had such a long conversation about all this …*

It was also a source of satisfaction because it gave them a sense of purpose:

*And (Female gender, age missing, spouse) I’m glad I did it. If it can help other families, that’s perfect! It’s important to move on!*

Honoring the memory of the deceased was another way to ease the pain and recover (29/39, 74%).

*(Female gender, age 59, spouse) People told me that the ceremony made them feel happy. […] That’s one of the key milestones that was very helpful, like an accomplishment. I feel like I accomplished something with this ceremony.*

1. *Social and emotional support*

The second type of facilitating factors refers to the social and emotional support provided by the medical team, therapists, association and family.

It can take the form of practical help, including information about death and bereavement, and emotional support, allowing the individual to feel valued, understood, and surrounded.

According to some of the participants, being informed of the outcome (i.e., the inevitable death) and how death will occur (i.e., what the final steps will be) allows one to be better prepared for the loss and, ultimately, to better adjust after death. More specifically, the quality and clarity of the communication provided by the medical team helped to prepare for loss. In fact, the gradual explanation of the course of the illness helped the bereaved to anticipate the final hours (23/39, 59%).

*(Female gender, age 50, daughter) After 5 months we knew the outcome, so we had time to prepare for the last hours. […] they took me aside and explained everything to me with kindness.*

Those who received clear information about first signs of death faced death with more anticipation and could gather people around them. This made death less brutal and they felt less abandoned at this critical moment (12/39, 31%).

*(Female gender, age 48, daughter) … the signs that could alert us a little bit and therefore we were a little bit more alert and indeed in the last hour … I managed to call my sons, well my ex-husband who drove the children to the hospital.*

After the death, feeling supported by the funeral team was also reported as a significant help (15/39, 38%). At the time of the death of the deceased, the relatives expressed their mental confusion and inability to make decisions, which is why the help and support of the funeral professionals was important to prevent drowning at this point. In addition, step-by-step leaflets on what to do and where to get help were appreciated.

*(Female gender, age 26, daughter) They had a leaflet, or something you could find online, anyway there are some summary documents and they explain everything that needs to be done.*

Emotional support is the second type of social support that facilitates grief.

First and foremost, support from friends, family and close relationships was reported as the most helpful. Respondents insisted on feeling listened to and surrounded. This allows them to share emotions, avoid running thoughts and isolation (14/39, 36%).

In contrast, a few relatives (10/39, 26%) had a preference for consulting psychotherapists or an organization specialized in the field. They expressed that they were afraid of adding to the pain of their loved ones. They felt more supported by mourning « experts ».

*(Female gender, age 66, spouse) I only called when I was in good shape, […] they did everything for me. I didn’t want to cause them more trouble, or anxiety. […] When you love someone and you try everything to make them feel better. The last thing you want to know is all this. I mean, I didn’t want them to feel like they did everything for nothing. But I needed something else. […] With Solange, my counsellor, I could call her anytime. […]she knew how to translate my feelings and reassure me … she understood everything. […] She is a professional, she would tell me: what you are experiencing is completely normal!*

Several attitudes emerged regarding the type of therapist to consult. Some respondents expressed their wish to see the psychologist they met in the hospital or palliative care unit, because he/she already knew them and the deceased:

*(Female gender, age 36, daughter) the connection I had with the psychologist in (name of the hospital) helped me a lot because she knew my mum from the beginning.*

Opinions differed as to whether they preferred to be contacted immediately after death or a few months later (from 3 to 12 months), in order to have some time for starting adjustment.

The role of associations was also mentioned. By talking with peers, relatives felt understood and were able to compare and share their experiences.

Finally, most participants (26/39, 67%) reported how grateful they were for the support received from the palliative care team. In the acute period before death, the flexibility and empathy shown by the team were important comforting factors.

More specifically, participants commented on the following:

- the empathetic listening and attitude: *(Female, age 71, daughter) I am very grateful to this place for their behavior and the way they do everything, with great humanity, and great listening.*
- the flexibility of the organization(*Female, age 59, spouse*) *I slept there the whole week and my son-in-law and my daughter-in-law and my daughters were also there … It was great. And that helped me a lot, a lot. I think for the first few months. It helped me a lot.*
- the attentive presence: *(Female gender, age 59, spouse) they literally open their arms to us, and they had that attitude even with the family, and also with visiting friends …*
- and follow up contacts: *(Female gender, age missing, spouse) And then I got their letter, and even a phone call from the palliative care unit, and frankly that was top notch! Without that help, I would not have been able to do it.*

##### Theme 4: Suggestion made by the bereaved for better coping with their grief

The last theme was the formulation of recommendations by the bereaved (in response to a question raised by the interviewer and spontaneously formulated in some cases) for dealing with the loss before and after the death. In relation to the elements described above as aggravating or facilitating factors, 5 recommendations emerged from the analysis: improving the communication, developing education about death and grief, maintaining contact, offering psychological support, and choosing the right time to make contact.

1. *Improve communication with the healthcare team*

As far as communication skills are concerned, participants want a mix of empathy, transparency and holding back (35/39, 90%).

Empathy rhymes with “finding the right words” and compassionate listening. It is especially important at specific times, such as diagnosis, transition to palliative care, and death.

*(Female gender, age 50, daughter) There are words to say it. Delicate words and silences, leaving the individual to realize for himself …*

Transparency in the information communicated includes the banishment of complicated speech that could leave room for inappropriate hope, so that relatives can begin to prepare themselves for the inevitable:

*(Female gender, age 35, daughter) Be more realistic and tell the truth without hesitation. then it will be easier to anticipate, prepare and cope with what comes next.*

Holding refers to guiding the relatives by taking the initiative. In fact, in some acute periods, the relatives feel confused and need to be guided by the hand:

*(Female gender, age 66, spouse) It’s up to the health professionals to guide us […] Everything has to be brought up and put in the person’s hand because sometimes you don’t know or don’t have enough strength to look for it …*

1. *Developing education about death and mourning*

The relatives expressed how overwhelmed they felt after the death (30/39, 77%). As a result, several suggestions were made to help the bereaved prepare for the post-mortem period and the onset of mourning.

One suggestion was to develop a comprehensive leaflet with to-do lists and useful numbers. This would help to deal with the many administrative issues:

*(Male gender, age 75, partner) Yes, I think it would be good to develop a booklet with all the procedures and contact details. It’s easy to forget what needs to be done. It will be really useful!*

The moment of the death of the loved one’s triggers anxiety that could be alleviated if it were anticipated. Relatives suggested that explanations and information should be given in advance:

*(Female gender, age 48, daughter) One night we were very anxious, my sister and I, because we were wondering how she was going to die? how people die? do they stop breathing? do they have a rattle? do they change color? are there any warning signs? […] These are silly questions […] but they would have really helped us. […] Explaining to people what the end of life is like, what it might look like. […] Talking about the last minutes, the last days.*

The mourning process also remains taboo and explanations are lacking. It is recommended that information about the mourning process be widely disseminated, in the form of a leaflet, an audio-guide or an internet website. This type of information should be easily accessible in the family room:

*(Female gender, age 50, daughter) Yes, a link to a website or something where you can log on when you want and when you are ready. Some people will log on two days later, others will do it after the funeral. And at least it’s not intrusive. Everyone gets what they need when they need it.*

1. *Maintaining boundaries*

The first weeks of mourning are characterized by a strong sense of abandonment, according to relatives.

Several expressed their wish to maintain contact with the care team, with whom they feel very close, some even calling them “family” (34/39, 87%) It was also suggested to organize a follow-up visit to the palliative care unit. However, most of the families do not feel able to ask for this to happen. The initiative has to come from the hospital team:

*(Female gender, age 55, spouse) Yes, it’s not a bad idea for the unit to follow up with families, but there is probably a hierarchy of who needs to be called first. If they know that the person is well surrounded, she/he will probably need less help than someone who is totally alone; so maybe a stronger presence is needed for people who are alone, and according to their availability, they should call more lonely people than those who are very well surrounded.*

1. *Provision of psychological support*

Psychological support for caregivers during the patient’s treatments and after the death was widely recommended (35/39, 90%).

The organization of discussion groups to share experiences of care and mourning was recommended:

*(Female gender, age 50, daughter) From time to time it would be nice to share things in a dedicated space, a coffee …’it’s difficult to go to the hospital every day, to put your life on hold, to forget the guilt and regret of everyday life and all that you can’t do anymore, we’re not superheroes. […]to know that we are not alone. […]putting ourselves on someone’s shoulder, letting go of the stress that is inside us …*

1. *Timing for follow-up contacts*

Relatives generally agree on the fact that they would probably not have been able to answer or respond to any contact from the hospital during the first 3 months after the death, because they were too busy and shocked (30/39, 77%).

Follow-up calls, visits and cards, might be better received if sent 3 months after the death of the loved one. The relatives recommended that these contacts should be regular and last for at least a year; several (6/39, 15%) suggested a frequency of once a month.

*(Male gender, age 51, son) But I am not sure that only one call would do the job. Why not offer the family or the remaining children several interviewees to discuss the death? […] I think it has to be a long-term process, including several sessions. Several phone calls.*

### Discussion

This study enabled to collect the grief experience of relatives with prolonged grief symptoms (ICG > 25) and to have access to their recommendations. The analysis enabled us to identify 4 themes: (1) the grief experience, (2) the aggravating factors, (3) the facilitating factors of the grief experience, and (4) the suggestions that the bereaved may have. Honest and empathic communication, an administrative leaflet, a mourning leaflet, a link with the team several months after the death were the main recommendations of the responding relatives.

The results underline the validity of Prigerson’s criteria for prolonged grief disorder of the DSM5-TR. Subjects accurately described separation distress, intense longing for the deceased, preoccupations with thoughts about the deceased, intense emotional pain, and intense loneliness. The criterion of dysfunction (“The disturbance causes clinically significant distress or impairment in social, occupational, or other important areas of functioning”) was missing, due to the qualitative design of the study, which did not cover all the diagnostic aspects.

Risk factors for prolonged grief has been recently reported (Buur et al. 2024). Our study was in line with some of them but our study found that the participants highlighted their lack of information about the concrete course of death. While the emphasis was rightly placed on the need of certain people to benefit from psychological monitoring, we must not neglect the importance of illuminating this need for concrete information that certain participants reported. This is a more specifically medical and/or nursing mission.

Our study showed that simple interventions may improve the lives of bereaved people while must be adapted to family dysfunction. Family dysfunction is important to consider in the level of psychosocial morbidity, with greater levels present in families whose functioning as a group was poorer (Schuler et al. 2017). Prolonged grief disorder therapy combining loss-focused components (accepting the reality of the death and changing the relationship with the deceased) and components focused on the future without the deceased, showed efficacy (Shear et al. 2005). Several cognitive behavioral therapies have also been reported with positive effects in various settings (Litz et al. 2014; Treml et al. 2021). A randomized trial that included bereaved individuals at risk for prolonged grief, 8 months after loss, reported significant efficacy of a cognitive-behavioral therapist-assisted, internet-delivered intervention for prolonged grief, anxiety, depression, and PTSD (Litz et al. 2014). Internet-based cognitive-behavioral grief therapy (ICBGT) has also been tested specifically for post-loss people bereaved by suicide, and reported reduction in symptoms of common grief reactions and depressive symptoms after suicide (Treml et al. 2021). These studies were in line with our results showing that staying in touch with the bereaved was one of their most important needs to better live the absence of their relative.

To fulfill these wishes and to better cope with their grief, relatives reported several recommendations for the time before the loss. They requested full transparency from the medical team about the course of the disease, with an empathic approach, in order to better prepare themselves for the death of their loved one. These results were in line with researches showing that preparation improves adjustment to bereavement (Hebert et al. 2006; Wen et al. 2022), with the interesting question of how to operationalize what preparation would be (Harrop et al. 2020). They called also for open communication about the symptoms of the last minutes of life. In their experience, the anxiety seemed to be related to the lack of knowledge about what could happen at the time of death. All subjects expressed a sense of the brutality of the death, of not being able to talk about it while their loved ones were in palliative care. This was also reported in relatives of critically ill patients where the communication with the patient was impaired and where poor communication with physicians and lack of contact with their loved one were reported as independent factors that increased the intensity of grief reactions in relatives 6 months after the loss (Kentish-Barnes et al. 2015). Family conference to prepare relatives for the impending death, a room visit to provide active support, and a meeting after the patient’s death to offer condolences and closure was shown to be effective in reducing prolonged grief reactions 6 months after the patient’s death (Kentish-Barnes et al. 2022).

After death, relatives have to face both administrative and psychological difficulties, as well as an intense distress. They asked of an administrative leaflet and the explanations of the different stages of grief were among their requests. This leaflet has been used with relatives of intensive care unit patients and has been shown to be effective (Lautrette et al. 2007). Therefore, it seems important to us to consider that preparation is different from information, and that it would be necessary to investigate the subjective elements that really enable relatives to prepare for grief and not only for death. Talking about the afterlife while the patient is still alive was one of their requests (Moon 2016; Nielsen et al. 2016; Tsai et al. 2016).

Bereavement programs varied widely, and their effectiveness in palliative care has been poorly documented. Studies of reporting practices are available (Kusano et al. 2012; Morris and Block 2015). Physicians were more prone to respond to a phone call from families than to be proactive in a bereavement program (Kusano et al. 2012). Only one-third of physicians were active in sending a condolence letter or organizing a family meeting (Kusano et al. 2012). It was felt important that the medical team approached relatives rather than waiting for them to ask for help (Chiu et al. 2010). Asking the medical team for help after the death of a loved one remains very difficult, even impossible for some. A phone call or a letter after the death can be helpful, but it is important to be careful about the timing: the bereaved pointed out that it is more positively received at least 3 months after the death and that it should be individualized according to the relatives (Kentish-Barnes et al. 2017). These elements raise the question of a systematic phone call in addition to a letter, which could be tested as an intervention. A phone call would provide a warmer contact and could facilitate the request for help.

The results of this study showed that a multifaceted program including empathic and transparent information about the course of cancer, information about how the patient will die, especially in the last minutes of life, administrative and bereavement leaflets, and telephone contacts from the palliative team could be proposed for designing an intervention study.

### Limitations

This study has several limitations. First, regarding the sample, the circumstances of death are limited in that the deceased are all cancer patients who died in palliative care units. The results of the study should not be generalized to other circumstances. Second, only some of the relatives with prolonged grief symptoms participated in the interview. It is legitimate to wonder whether those who did not participate may not have been the most dissatisfied with the medical care or the most in difficulty during their grieving process and did not want to relive their traumatic experience. In particular, our study reported that most of the relatives who had previously experienced palliative care hospitalization did not participate in the study. Third, we used the ICG questionnaire rather than the Prolonged Grief-13 (PG-13), while the latter is now the most widely use diagnostic questionnaire. Fourth, we measured prolonged grief symptoms rather than prolonged grief disorder. Fifth, the last part of the interviews was conducted during the SARS-CoV2 pandemic; it is necessary to question how the pandemic situation might have influenced the grieving process. Even if in the quantitative part of this study the prolonged grief score was not impacted by the lockdown period, we cannot negate how the relatives described this period and how anxiety and depression states influenced their bereavement (Garrouste-Orgeas et al. 2023).

## Supporting information

Flahault et al. supplementary material Flahault et al. supplementary material

## Data Availability

The datasets used and/or analyzed during the current study are available from the corresponding author on reasonable request.

## Supplementary material

The supplementary material for this article can be found at https://doi.org/10.1017/S1478951524001068.
