# Supplementary material for: Healing grief: Insights from relatives of cancer patients with prolonged grief. The FamiLife multicenter qualitative study
Source: Palliat Support Care. 2024 Oct 31;23:e13. doi: 10.1017/S1478951524001068 (PMC13166286; doi:10.1017/S1478951524001068)
Supplement: Flahault et al. supplementary material [file S1478951524001068sup001.docx]

**Healing Grief: insights from cancer patients’ relatives with prolonged grief. The FamiLife multicenter qualitative study**

Cécile Flahault, PhD, Léonor Fasse, PhD, Laetitia Veber, Msc, Marie Sonrier, Msc, Marie Annick Leborgne, Msc, Dominique Michel, MD, Véronique Marché, MD, Anne Vanbésien MD, Adrien Evin, MD, Nicolas Pujol, Msc, Laure Copel, MD, Willeme Kaczmarek, MD, Sylvie Kirsch, Msc, Catherine Verlaine, MD, Virginie Verliac, MD, Emmanuel Delarivière, MD, Virginie Fosset-Diaz, MD, Virginie Guastellas, MD, Véronique Michonneau-Gandon, MD, Ségolène Perruchio, MD, Gaelle Ranchou, MD, Laurence Birkui de Francqueville, MD, Cécile Poupardin MD, Licia Touzet MD, Carmen Mathias MD, Alaa Mhalla MD, PhD, Guillaume Bouquet MD, Bruno Richard MD, PhD, Dominique Gracia MD, Florent Bienfait MD, Stéphane Ruckly, Msc , Jean François Timsit, MD, PhD, Maité Garrouste-Orgeas, MD.

**Supplement material**

**e-Table 1**. Characteristics of the recruitment in 26 palliatives care units.

|  |  |  | **Quantitative analysis Familife study**  **(Garrouste-Orgeas et al. 2023)** | | | | | **Qualitative analysis** | | | |
| --- | --- | --- | --- | --- | --- | --- | --- | --- | --- | --- | --- |
| **Centres** | **First inclusion** (YYYY/MM/DD) | **Last inclusion** (YYYY/MM/DD) | **Relatives**  **included** | **Relatives of alive patients** | **Relatives refusal** | **Relatives lost of follow-up** | **Relatives analyzed** | **ICG > 25** | **Lost of follow-up** | **Relatives refusal** | **Relatives interviewed** |
| 1 | 2019/01/28 | 2020/01/30 | 118 | 17 | 6 | 18 | 77 | 15 | 10 | 1 | 4 |
| 2 | 2019/03/04 | 2019/03/24 | 2 | 0 | 0 | 0 | 2 | 2 | 1 | 0 | 1 |
| 3 | 2019/04/01 | 2020/01/04 | 59 | 1 | 7 | 7 | 44 | 14 | 9 | 2 | 3 |
| 4 | 2019/02/25 | 2020/01/25 | 36 | 0 | 2 | 7 | 27 | 13 | 9 | 0 | 4 |
| 5 | 2019/04/26 | 2019/07/08 | 10 | 2 | 0 | 0 | 8 | 4 | 3 | 0 | 1 |
| 6 | 2019/04/15 | 2020/02/04 | 31 | 0 | 3 | 1 | 27 | 4 | 2 | 0 | 2 |
| 7 | 2019/03/01 | 2020/01/30 | 23§ | 3 | 4 | 2 | 12 | 8 | 6 | 2 | 0 |
| 8 | 2019/03/06 | 2019/06/27 | 26\|\| | 9 | 0 | 4 | 12 | 0 | 0 | 0 | 0 |
| 9 | 2019/03/12 | 2019/11/22 | 26 | 1 | 1 | 2 | 22 | 13 | 8 | 3 | 2 |
| 10 | 2019/09/03 | 2020/02/06 | 93¶ | 9 | 7 | 6 | 67 | 20 | 11 | 5 | 4 |
| 11 | 2019/02/19 | 2019/12/12 | 24 | 5 | 4 | 0 | 15 | 6 | 4 | 1 | 1 |
| 12 | 2019/03/14 | 2020/01/31 | 21 | 0 | 2 | 1 | 18 | 7 | 4 | 1 | 2 |
| 13 | 2019/02/27 | 2020/01/06 | 19 | 2 | 4 | 2 | 11 | 5 | 4 | 0 | 1 |
| 14 | 2019/04/15 | 2019/07/04 | 13 | 10 | 0 | 0 | 3 | 2 | 0 | 0 | 2 |
| 15 | 2019/03/05 | 2019/10/31 | 44 | 3 | 6 | 5 | 30 | 5 | 3 | 1 | 1 |
| 16 | 2019/04/02 | 2019/06/14 | 18 | 0 | 2 | 4 | 12 | 8 | 7 | 1 | 0 |
| 17 | 2019/03/19 | 2019/07/25 | 30 | 0 | 3 | 1 | 26 | 6 | 4 | 1 | 1 |
| 18 | 2019/03/15 | 2019/10/21 | 22 | 0 | 2 | 1 | 19 | 7 | 6 | 1 | 0 |
| 19 | 2019/02/22 | 2020/02/05 | 72 | 15 | 6 | 2 | 49 | 22 | 16 | 3 | 3 |
| 20 | 2019/03/19 | 2019/08/22 | 18 | 0 | 1 | 0 | 17 | 8 | 7 | 0 | 1 |
| 21 | 2019/03/27 | 2019/04/11 | 8 | 3 | 0 | 0 | 5 | 2 | 1 | 0 | 1 |
| 22 | 2019/01/31 | 2020/01/23 | 87 | 14 | 3 | 7 | 63 | 19 | 13 | 3 | 3 |
| 23 | 2019/05/09 | 2019/09/08 | 12 | 0 | 1 | 1 | 10 | 2 | 1 | 0 | 1 |
| 24 | 2019/06/05 | 2020/01/10 | 10 | 2 | 0 | 1 | 7 | 0 | 0 | 0 | 0 |
| 25 | 2019/04/04 | 2019/10/08 | 17 | 0 | 1 | 1 | 15 | 0 | 0 | 0 | 0 |
| 26 | 2019/07/27 | 2020/01/16 | 15 | 0 | 1 | 1 | 13 | 7 | 6 | 0 | 1 |
| Overall | 2019/01/28 | 2020/02/06 | 854 | 96 | 66 | 74 | 611** | 199 | 136†† | 24‡‡ | 39§§ |

Protocol violation by relatives: § (n=2), || (n=1), ¶ (n=4). Of the 611** relatives analyzed, 608 remained in the principal analysis, due to the lack of Inventory Complicated Grief questionnaire for 3 relatives

†† Relatives who did not sent back their informed consent

‡‡ Relatives who expressed their refusal either by phone or in writing
